# Necrosis targeted radiotherapy with iodine-131-labeled hypericin to improve anticancer efficacy of vascular disrupting treatment in rabbit VX2 tumor models

**DOI:** 10.18632/oncotarget.3679

**Published:** 2015-03-29

**Authors:** Haibo Shao, Jian Zhang, Ziping Sun, Feng Chen, Xu Dai, Yaming Li, Yicheng Ni, Ke Xu

**Affiliations:** ^1^ Department of Radiology, The First Hospital of China Medical University, Shenyang, China; ^2^ Laboratory of Translational Medicine, Jiangsu Provincial Academy of Traditional Chinese Medicine, Nanjing, China; ^3^ Radiation Medical Institute, Shandong Academy of Medical Sciences, Jinan, China; ^4^ Department of Imaging & Pathology, Theragnostic Laboratory, University of Leuven, Leuven, Belgium

**Keywords:** necrosis, targeted radiotherapy, hypericin, vascular disrupting treatment

## Abstract

A viable rim of tumor cells surrounding central necrosis always exists and leads to tumor recurrence after vascular disrupting treatment (VDT). A novel necrosis targeted radiotherapy (NTRT) using iodine-131-labeled hypericin (^131^I-Hyp) was specifically designed to treat viable tumor rim and improve tumor control after VDT in rabbit models of multifocal VX2 tumors. NTRT was administered 24 hours after VDT. Tumor growth was significantly slowed down by NTRT with a smaller tumor volume and a prolonged tumor doubling time (14.4 *vs*. 5.7 days), as followed by *in vivo* magnetic resonance imaging over 12 days. The viable tumor rims were well inhibited in NTRT group compared with single VDT control group, as showed on tumor cross sections at day 12 (1 *vs.* 3.7 in area). High targetability of ^131^I-Hyp to tumor necrosis was demonstrated by *in vivo* SPECT as high uptake in tumor regions lasting over 9 days with 4.26 to 98 times higher radioactivity for necrosis versus the viable tumor and other organs by gamma counting, and with ratios of 7.7–11.7 and 10.5–13.7 for necrosis over peri-tumor tissue by autoradiography and fluorescence microscopy, respectively. In conclusion, NTRT improved the anticancer efficacy of VDT in rabbits with VX2 tumors.

## INTRODUCTION

Vascular disrupting treatment (VDT) has been considered a potentially important option for cancer therapy [1-3]. Vascular disrupting agents (VDAs) target the established tumor blood vessels rapidly and selectively, resulting in widespread ischemia and necrosis of the tumor [4, 5]. However, in clinical trials, minimal objective tumor responses were noted [1, 3, 6]. Experimental and clinical observations have demonstrated significant but transient reductions in tumor perfusion [6, 7]. A rim of viable tumor cells survived at the periphery, despite more than 90% of the central region of tumor experiencing rapid necrosis after administration of VDAs [8]. Therefore, these viable tumor cells are essential to tumor recurrence and metastasis. Re-growth and metastasis instead of primary tumor growth were believed to be the key contributors to the reduced overall survival benefit of cancer patients [9].

Efforts have been made to overcome the resistance of tumors to VDAs. Some treatment modalities such as radiotherapy, chemotherapy, immunoradiotherapy, or antiangiogenic drugs have been complementarily used to treat the surviving tumor rim [10-13]. Although short-term enhanced tumor controls were observed in some of the studies, no substantial progress has been made in overcoming viable rim-induced cancer recurrence to date.

**Figure 1:**
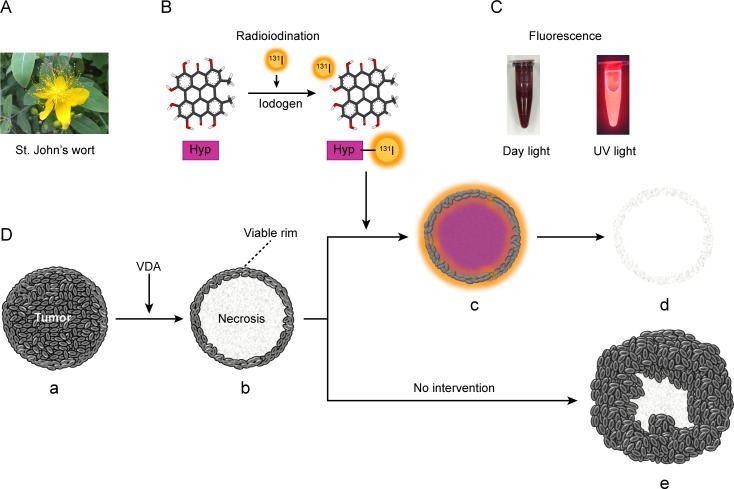
St. John's wort and Necrosis Targeted Radiotherapy (NTRT) **A**. St. John's wort is a yellow-flowering plant of the genus *Hypericum,* which can be purified into hypericin (Hyp). **B**. Hyp has the properties of being highly radioiodinated with an iodogen method. **C**. Hyp gives red fluorescence under UV light. **D**. Schematic diagram of NTRT. After vascular disrupting treatment, the tumor (a) becomes centrally necrotic on a large scale accompanied by a thin viable rim (b) because of the different blood supplies. Intravenously administered ^131^I-Hyp may selectively reside in the necrosis and deliver highly intensive beta particles that efficiently kill peripheral viable malignant cells (c). Finally, the whole tumor may be destroyed (d). By contrast, the viable rim will eventually progress into recurrence if no effective intervening measures are taken (e).

The necrosis-targeted therapeutic strategy was proposed based on the studies of necrosis-avid contrast agents (NACAs) [14]. Hypericin (Hyp), a natural small molecular compound extracted from St. John's wort (Hypericum perforatum), was recently recognized as an NACA with powerful necrosis affinity [15-18]. In addition to its reported anti-viral, anti-tumor and antidepressant activities [19], Hyp has other two properties: one is photosensitivity, which permits the application of Hyp in fluoroscopy and photodynamic therapy [20-21]; the other is that Hyp can be radioiodinated promptly, efficiently and persistently [15-18, 22]. In previous preclinical studies, radioiodinated Hyp has been used in the diagnosis of myocardial infarction and discrimination of therapeutic necrosis from residual tumors, with target-to-nontarget ratios of approximately 30–80, which are much higher than those with the ever-discovered specific agents [16-18]. Thus, we assume that iodine-131-labeled hypericin (^131^I-Hyp) may selectively accumulate in tumor necrosis induced by VDAs and deliver high-energy beta particles to destroy the surrounding remaining malignant viable cells. The working principle of the novel necrosis-targeted radiotherapy (NTRT) is illustrated in Fig. 1.

**Figure 2:**
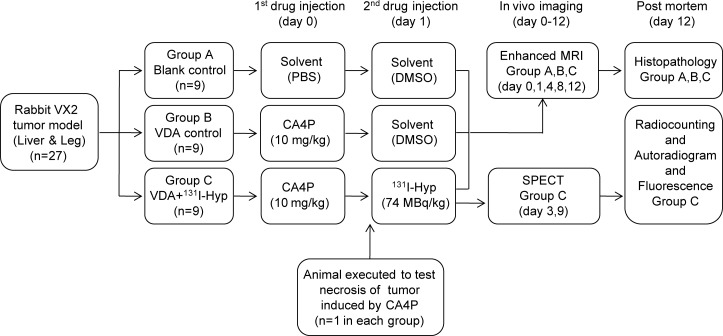
Flow diagram of the experimental procedures in rabbits with implanted bifocal VX2 tumors on both the liver and right leg (VDA, vascular disrupting agent; Hyp, hypericin; PBS, phosphate-buffered saline; DMSO, Dimethyl sulfoxide).

The purpose of this study was to validate the new therapeutic strategy with NTRT in rabbit VX2 tumor models. Based on experiments in small rodents [23-25], we show for the first time that such medium-sized animal models can be used to verify the properties of NTRT. Furthermore, a more clinically relevant multifocal tumor model (in both the muscle and liver) was used to imitate the primary tumor coexisting with metastasis, which could simulate the conditions of clinical patients receiving VDT [1, 3, 6, 7, 10-13].

## RESULTS

### General conditions

The rabbit models of muscle and liver VX2 tumors were successfully established in all animals enrolled in the present study. All of rabbits survived the anesthesia, surgical procedures, drug injection, tumor growth and *in vivo* imaging sessions. Tumor necrosis was successfully induced with CA4P in all of the rabbits in groups B and C. ^131^I-Hyp was formulated with a labeling rate of 99.6% and was successfully injected intravenously in all of the rabbits in group C.

### Tumor changes on dynamic MR imaging

**Figure 3:**
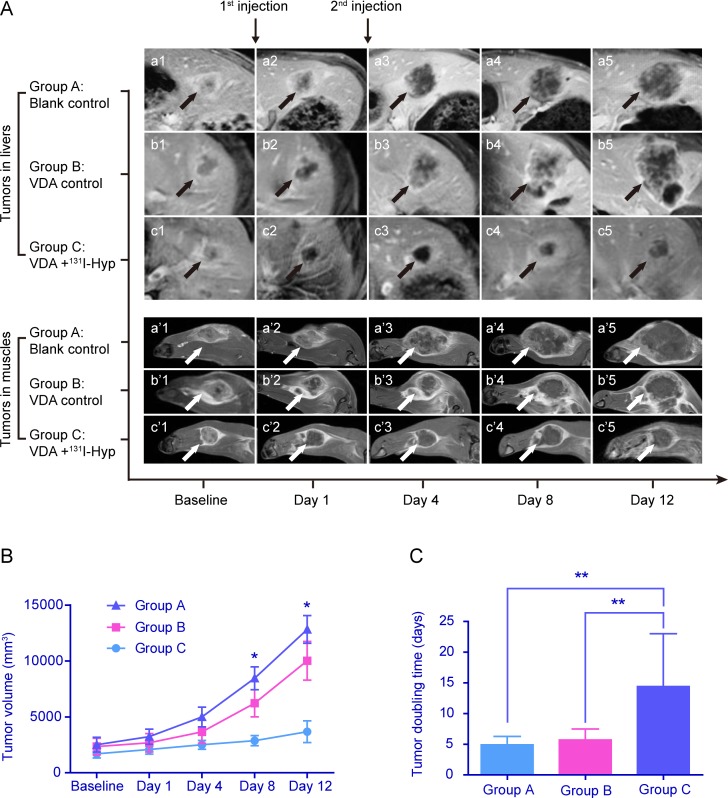
Dynamic MRI following up of representative tumor-bearing animals in each group **A**. The enhanced T1-weighted MR images (a1-c1, a'1-c'1) at baseline showed that the oval tumors on the liver (black arrows) and leg (white arrows) in each group were similar in size and enhanced to the same extent. Twenty-four hours after the first injection, the enhancement was shut down in the center of the tumors in groups B and C (b2, b'2 and c2, c'2) compared with the tumors in group A (a2, a'2). By the second injection, the tumors in group C became stable in volume and were maintained as non-enhanced centrally throughout the follow-up period (c3-c5, c'3-c'5). By contrast, the tumors in groups A and B grew faster with larger tumor volumes (a3-a5, a'3-a'5 and b3-b5, b'3-b'5) than those in group C. The central non-enhanced necrosis appeared with thicker, irregularly enhanced rims in group B (b3-b5, b'3-b'5). **B**. The growth curve of tumors at different time points showed no difference in tumor volume among the 3 groups at baseline. The tumors grew fastest in group A, and tumor growth was the lowest in group C. The tumor volumes were significantly larger in group A and B than those in group C at day 8 and day 12. **C**. The mean TDT in group C was significantly longer than that in group A and B. *p < 0.05, ** *p* < 0.01.

The tumors in the livers (Fig. 3A, *a-c*) and muscles (Fig. 3A, *a'-c'*) were clearly shown on contrast-enhanced T1-weighed MR images and demonstrated similar changes on imaging among the different groups. At baseline, the tumors in each group appeared to have similar sizes, round or oval shapes and moderate enhancement (Fig. 3A, *a*1-*c*1, *a'*1*-c'*1). Twenty-four hours after the first injection of CA4P, the enhancement was sharply decreased in the center of the tumors in groups B and C, accompanied with residual enhanced rims (Fig. 3A, *b*2, *c*2, *b*'2, *c*'2). The corresponding histological results verified that the imaging changes were caused by ischemic central necrosis and residual peripheral tumor blood vessels. There was no change in group A on MR images between the baseline and 24 h (Fig. 3A, *a*1, *a*2, *a'*1, *a'*2). By the second injection of ^131^I-Hyp, the tumor growth in group C was well inhibited and maintained a stable in size until the endpoint (Fig. 3A, *c*3-*c*5, *c'*3-*c'*5). The tumors showed no enhancement with only thin and intermittently enhanced rims, findings that were speculatively attributed to beta irradiation of tumors by ^131^I-Hyp as supported by SPECT images. By contrast, the tumors in groups A (Fig. 3A, *a*3-*a*5, *a'*3-*a'*5) and B (Fig. 3A, *b*3-*b*5, *b'*3-*b'*5) grew rapidly with the tumor size progressively becoming larger than that in group C. Irregularly enhanced thick rims suggestive of viable tumor tissue were shown in group B at later time points (Fig. 3A, *b*3-*b*5, *b'*3-*b'*5).

### Tumor volume and tumor doubling time

As shown in Fig. 3B, the tumor volumes displayed no difference among the groups at baseline (2512±655, 2343±738 and 1694±377 mm^3^ in group A, B and C, *p* > 0.05). The tumors grew rapidly in groups A and B but slowly in group C, with no significant difference in tumor volumes among the groups at day 1 (3227±684, 2681±823 and 2087±414 mm^3^ in groups A, B and C, respectively; *p* > 0.05) and day 4 (4990±882, 3656±1033 and 2502±397 mm^3^ in groups A, B and C, respectively; *p* > 0.05). With further follow up, the tumor volumes were significantly larger in groups A and B than those in group C at day 8 (8452±1021, 6220±1214 and 2872±457 mm^3^ in group A, B and C, respectively; *p* < 0.05; A vs. B, *p* > 0.05; A vs. C, *p* < 0.05; B vs. C, *p* < 0.05) and day 12 (12823±1237, 10012±1730 and 3670±968 mm^3^ in groups A, B and C, respectively; *p* < 0.05; A vs. B, *p* > 0.05; A vs. C, *p* < 0.05; B vs. C, *p* < 0.05). TDT (Fig. 3C) was significant longer in group C (14.4±8.6 days) than that in group A (4.9±1.4 days) and group B (5.7±1.8 days) between day 12 and baseline (*p*<0.01 among the 3 groups, A vs. B, *p* > 0.05; A vs. C, *p* < 0.01; B vs. C, *p* < 0.01).

### Macroscopic histopathology and area of viable rim

**Figure 4:**
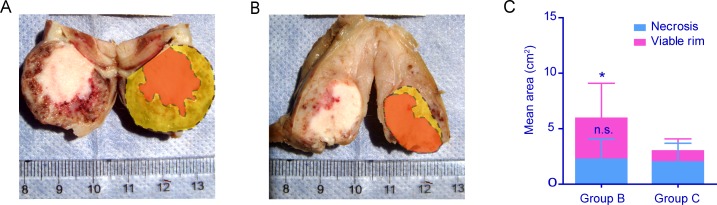
Macroscopic view of split tumor and measurement of viable rim area **A** & **B**. The macroscopic photos showed that central necrosis, viable tumor rim and peritumoral tissues could be easily discriminated on the representative split tumor specimen in group B and C. The tumor in group B (**A**) was bigger and with thicker viable rim significantly than that in group C (**B**) The tumor and central necorsis (orange area) was delineated by the software of Adobe Photoshop CS5 Extended 12.0. The area of viable rim (yellow area) was drawn from area difference of tumor and central necrosis. **C**. By quantitative analysis the mean area of viable rim in group B was 3.7 times larger than that in group C (*p < 0.05). There was no significance (n.s.) in area of central necrosis between the two groups. The whole tumor area in group B was 1.98 times larger than that in group C (*p < 0.05). This indicated that inhibition of viable rim led to better tumor control.

Macroscopically, central necrosis, viable tumor rim and peritumoral tissues could be easily discriminated on the resected cross-sections of the split tumor specimen in group B and C (Fig. 4). The tumors in groups B were significantly larger with thicker viable tumor rims than those in group C, which were consistent with MRI findings. By analysis of the Adobe Photoshop CS5 Extended 12.0 software, the mean gross tumor area in group B was 1.98 times larger than that in group C (5.94±4.96 vs. 3±2.76, *p* < 0.05). Furthermore, the mean area of viable rim was 3.7 times larger in group B than in group C (3.7±3.15 vs. 1±1.08, *p* < 0.05). However, the mean area of central necrosis was of no difference between the two groups (2.24±1.81 vs. 2±1.68, *p* > 0.05). It indicated that viable rim control by irradiation led to ideal whole tumor volume control.

### *In vivo* SPECT and postmortem gamma counting

**Figure 5:**
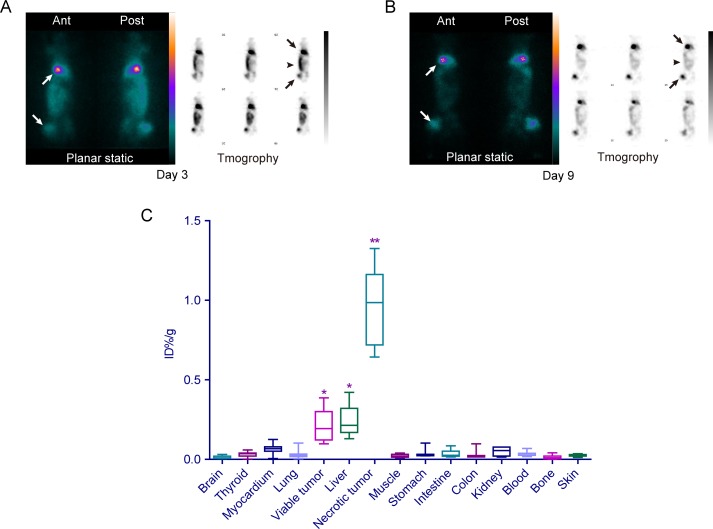
SPECT and gamma counting **A** & **B**. The planar static and tomographic images of SPECT at day 3 (**A**) and day 9 (**B**) showed the persistently strong radioactivity focused on tumor regions (white and black arrows) in the liver and right leg. Strong radioactivity also appeared in the intestinal region (black arrowheads) at day 3 (**A**) and was obviously weakened at day 9 (**B**) indicative of the excretion pathway for ^131^I-Hyp. **C**. Box and whisker plots quantitatively showed the bio-distribution of ^131^I-Hyp in different tissues of the animals in group C at day 12 (11 days after the injection of ^131^I-Hyp). **The injected dose per gram of necrotic tumor was significantly higher than that of the viable tumor, liver and other organs (*p* < 0.01). *The injected dose per gram of viable tumor and liver was significantly higher than that of other organs (*p* < 0.05).

Two days after injection of ^131^I-Hyp (day 3), the planar static and tomographic images showed strong radioactive concentration or hot spots at tumor regions (Fig. 5A, white and black arrows) on the liver and right leg of the animals in group C. Additionally, abundant radioactivity was detected in the intestinal tract (Fig. 5A, black arrowhead), which was considered a major excretion pathway of Hyp. Over time, the tumor regions showed a persistently high concentration of radioactivity, while ^131^I-Hyp was washed out from intestines. Compared with the images at day 3, those at day 9 (about one half-life of ^131^I) showed equally strong hot points at tumor regions (Fig. 5B, white and black arrows) but significantly reduced radioactivity at intestinal regions (Fig. 5B, black arrowhead). The tumor to intestine ratio of radioactive intensity at day 9 (18.8±7.3) was 5 times of that at day 3 (3.7±2.1).

The results of postmortem gamma counting at day 12 showed that the radioactivity in necrotic tumors was much higher than that in viable tumors and other organs (Fig. 5C). The radioactivity of necrotic tumors (0.98±0.24 ID%/g) was 4.26 times that of viable tumors (0.23±0.10 ID%/g, *p*<0.01) and 4.45 times that of the liver (0.22±0.10 ID%/g), followed by the lung (0.06±0.03 ID%/g), kidney (0.05±0.02 ID%/g), intestine (0.03±0.02 ID%/g), heart (0.03±0.02 ID%/g), stomach (0.03±0.02 ID%/g), thyroid (0.03±0.01 ID%/g), blood (0.03±0.01 ID%/g), colon (0.02±0.03 ID%/g), skin (0.02±0.01 ID%/g), muscle (0.02±0.01 ID%/g) and brain (0.01±0.01 ID%/g) (*p* < 0.01 for all organs compared with necrotic tumors). The radioactivity in viable tumors and the liver was also significantly higher than that in other organs (*p* < 0.01).

### Autoradiography and fluorescence microscopy

**Figure 6:**
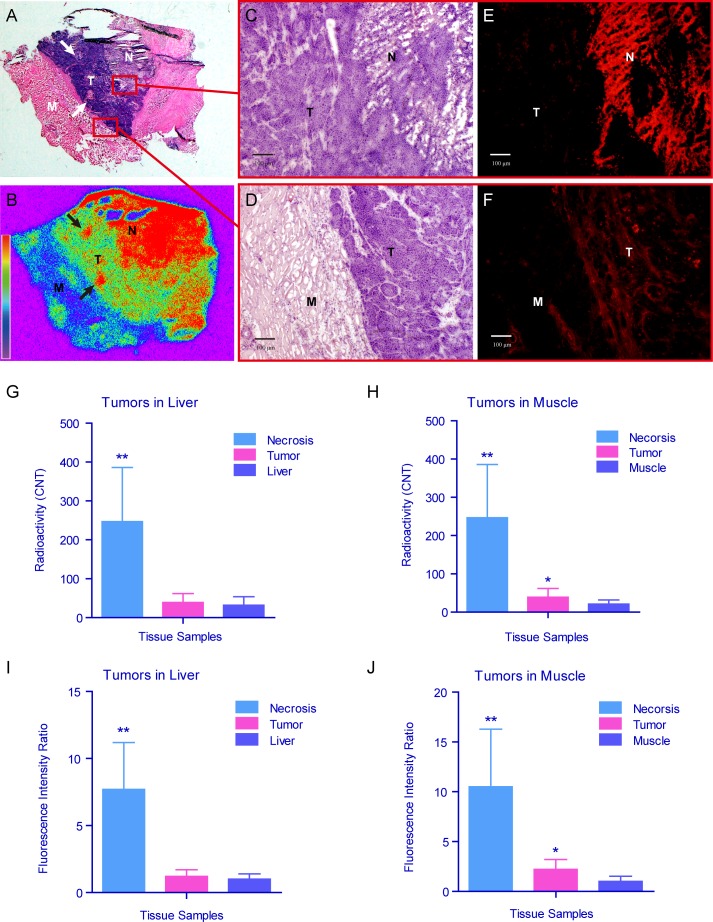
Autoradiography and fluorescence microscopy of the tumors in group C **A** & **B**. The tumor harvested from the leg of the rabbit in group C including necrosis, viable tumor rim and muscle was prepared as serial cryostat sections. The digital photographs of H&E (**A**) and corresponding autoradiography (**B**) sections showed the distribution of radioactivity intensity (necrosis>viable tumor>muscle). The dotted necrotic foci (black and white arrows) within the viable rim also revealed high intensity of radioactivity. The microscopic views were focused on the borders between necrosis, viable tumor rim and muscle (red frames). **C**-**F.** The light. (**C** & **D)** and corresponding fluorescence (**E** & **F)**. microscopic graphs showed the distribution of fluorescence intensity (necrosis>viable tumor>muscle). **G**-**J**. The quantitative analysis showed the radioactivity (**G** & **H)** and intensity of fluorescence (**I** & **J)** was significantly stronger and higher in necrosis than those in viable tumor and peri-tumor tissues (muscle and liver). ** The radioactivity and intensity of fluorescence was significantly higher in necrosis than those in viable tumor, liver and muscle (*p* < 0.01). *The radioactivity and intensity of fluorescence was higher in viable tumor than those in muscle (*p* < 0.05). (H&E, hematoxylin and eosin; Auto-Rx, autoradiography; N, necrosis; T, tumor, M, muscle).

The necrosis avidity of ^131^I-Hyp was validated postmortem using autoradiography and fluorescence microscopy. The animals only in group C were subjected to analyses. The tumors appeared nearly complete necrosis on macroscopic specimens. Only very thin viable rims could be seen in some cases. Tumors were sampled for autoradiography and fluorescence microscopy. Corresponding to the H&E sections (Fig. 6A), color-coded autoradiography (Fig. 6B) visually displayed radioactivity distribution among necrosis, viable rim and peritumoral tissue with clear borders. The central necrosis (red color) showed much stronger radioactivity than the viable tumor rim (green color) and peritumoral muscular tissue (blue color). A better proof of the necrosis avidity of ^131^I-Hyp was that dotted necrotic foci (white and black arrows in Fig. 6A and 6B) within viable tumors also appeared as strong radioactivity of red color. Quantitative analysis (Fig. 6G and 6H) showed the radioactive intensity of necrosis (247±139 CNT) was approximately 6.3 times higher than that of tumors (39±23 CNT, *p* < 0.01 vs. necrosis), 7.8 times higher than that of peritumoral liver (32±22 CNT, *p* < 0.01 vs. necrosis, *p* > 0.05 vs. tumor) and 11.7 times higher than that of peritumoral muscle (21±11 CNT, *p* < 0.01 vs. necrosis, *p* < 0.05 vs. tumor), with the ratios of necrosis/tumor/liver and necrosis/tumor/muscle as high as 7.7/1.2/1 and 11.7/1.8/1, respectively.

To visualize in greater detail the selective retention of ^131^I-Hyp in tumor necrosis, intratumoral distribution was analyzed by fluorescence microscopy. Necrosis, viable tumor and peritumoral tissues were distinguished by H&E staining (Fig. 6C and 6D), showing different patterns on microscopic images. To compare the fluorescence distribution, microscopy was focused on the borders between different tissues. Fluoromicroscopic images revealed distinct fluorescence intensity in necrosis and viable tumors, and peritumoral tissues (Fig. 6E and 6F), with necrosis/tumor/liver and necrosis/tumor/muscle ratios of 10.5/ 2.2/1 and 13.7/1.8/1, respectively (Fig. 6J and 6K).

## DISCUSSION

The new therapeutic strategy of complementary necrosis targeted radiotherapy (NTRT) was validated in the present study in rabbit VX2 tumor models mimicking a primary tumor at one site (e.g., muscle) with metastasis to another site (e.g., liver). The results indicated that the residual viable tumor rim around necrosis induced by a VDT could be well controlled by NTRT. To the best of our knowledge, therapeutics or NTRT specifically designed to treat a viable tumor rim after VDT has not yet been reported in rabbit VX2 tumor models.

Using the “soil-to-seeds” principle, such a sequential targeting theragnostic modality was proposed and evaluated in rodents in 2011 [23]. It was speculated that the proliferative parenchyma of mutational cancer cells (seeds) are intrinsically resistant to targeted therapeutic attacks, whereas the supportive stroma of connective tissues (soil) are more genomically stable and easier to target. To further develop this dual targeting anticancer approach, in the current study, we successfully verified its efficacy and safety in rabbit VX2 tumor models of virtual primary and metastatic tumors. Among patients who receive systematic targeting therapy, more than half of them had metastases besides the primary tumors [1, 3, 6, 7, 10-13]. Thus, this rabbit tumor model representatively imitated the clinical conditions of oncological patients. Furthermore, besides the therapeutic application, *in vivo* SPECT imaging might also allow the identification or monitoring of any known or unknown primary or metastatic lesions during treatment, follow up for therapeutic progress, and assessment of patient prognosis. Hence, NTRT has been regarded as a new targeting theragnostic approach [26-28].

The properties of NTRT were evaluated regarding two major aspects. The first is the strong targetability of ^131^I-Hyp to central necrosis of the tumor, a finding that was verified by *in vivo* SPECT, *ex vivo* gamma counting, autoradiography and fluorescence microscopy. The second is the superb tumoricidal efficacy of the combined use of CA4P and ^131^I-Hyp that was evidenced by the dynamic MR images and corresponding histopathology after only a single episode of NTRT administration.

An ideal targeting drug should have the properties of prompt target homing, persistent target residence and a high target-to-nontarget ratio. As a necrosis targeted agent, Hyp has shown a prominent targetability to tumor necrosis [16, 17, 23, 24, 26-28]. The targeting property of ^131^I-Hyp was verified in the present rabbit VX2 tumor model in two ways. The first is the detected gamma rays emitted from ^131^I, demonstrating *in vivo* as intensive and persistent hot-spots on tumor regions on SPECT scanning for at least 9 days and ex vivo as high gamma ray counts and as highly radioactive intratumoral necrotic zones on autoradiography at day 12. The second is the recorded coexisting red fluorescence emitted by Hyp in tumor necrosis. The highly concordant ^131^I and Hyp distribution in necrosis, tumor and peritumoral tissues also proved the stable conjugation of ^131^I and Hyp in the body. These results were consistent with those of previous studies in rats and mice. In a rat model of liver rhabdomyosarcoma-1 (R1), ^131^I-Hyp was found to accumulate in tumor necrotic regions at 3.13% ID/g, corresponding to a target-to-liver activity ratio of over 20 on day 8 after injection [23]. In mice carrying subcutaneous radiation-induced fibrosarcoma (RIF-1), a high concentration of ^131^I-Hyp was retained in tumor necrotic regions over 30 days, indicating long-term *in vivo* stability and targetability [23, 24].

The data from the above small and medium animal models demonstrated that ^131^I-Hyp is a potent agent for targeting necrotic tumors and appears superior to another radioiodinated agents used in tumor necrosis treatment (TNT). For instance, ^131^I-labeled chimeric monoclonal antibody (^131^I-mAb) was, until now, the only reported agent for TNT that targets a universal nuclear antigen histone [29, 30]. During TNT, only 0.001%–0.01% ID/g of ^131^I-mAb was found in treated tumors, resulting in a much lower targetability than that with ^131^I-Hyp [23, 31]. Furthermore, the poor pharmacokinetic profile, low tumor uptake owing to its large molecular mass, insufficient density of target antigens on tumor spots, as well as short serum half-life, limited the clinical use of ^131^I-mAb (32-34). ^131^I-Hyp could potentially expand the scope of TNT by introducing low-molecular-weight compounds without immunogenicity, bone marrow uptake and myelosuppression [16], important dose-limiting factors of TNT antibodies [27, 35]. Therefore, NTRT with ^131^I-Hyp could indicate substantial improvement in TNT.

A high and enduring concentration of ^131^I-Hyp in tumoral necrosis was responsible for the observed tumor control with NTRT. In group C, 0.98% ID/g of ^131^I-Hyp was found in necrotic tumors at day 12, a finding that is estimated to correspond to a cumulative dose of more than 2000 Gy. This radiation dose to the tumors was much higher than the minimum radiation dose of 50 Gy that was necessary to achieve a therapeutic response in most neoplasms [31]. Furthermore, ^131^I emitted β particles with a maximum penetration of 2.0 mm in tissues, a thickness that is far beyond the typical thickness of the remaining viable tumor of approximately 20–100 μm following CA4P treatment [27]. These observations can explain why NTRT in group C, compared with single administration of CA4P, well inhibited the tumor growth (about one-third of the volume in group B) and prolonged TDT (approximately 3 times that of group B) during the 12-day observation period. On the other hand, the tumor inhibition by a single dose of CA4P (group B) was not remarkable compared with the blank control (group A), results that were similar to those of reported clinical trials [11]. The lack of a significant tumor inhibitory effect in group B might be explained by hypoxia-induced tumor aggression following CA4P injection. With the regulation of hypoxia-inducible factor 1α (HIF-1α), expression of the angiogenic gene is activated to elevate the level of vascular endothelial growth factor, and endothelial progenitor cells are increased, subsequently promoting tumor neovascularization [36]. Additionally, the peripheral remnant tumor cells are supplied by nontumoral blood vessels and are oxygen saturated. Following the injection of CA4P, these tumor cells rapidly proliferate and become more sensitive to radiation, a condition that is readily suitable for NTRT with ^131^I-Hyp.

The biodistribution and excretion of ^131^I-Hyp in rabbits were documented in the present study, and the results were similar to previous results in rodents [23, 24, 27]. On SPECT imaging, besides the tumor regions, the intestines showed relatively high radioactivity at day 3 but not at day 9. Thus, the liver-bile-intestine axis was believed to be the excretion pathway of ^131^I-Hyp. However, relatively high gamma counts were still detected in rabbit liver at day 12, a result that was different from that in rodents [23]. The cause may be due to interspecies variations in uptake, metabolism and excretion of ^131^I-Hyp. Therefore, further studies in other animal species, particularly larger-sized animals such as canines or swine, are deemed necessary before the application in humans.

NTRT was well tolerated by all of the rabbits without the side effects commonly seen with other chemo- or radiotherapies. As described previously [17], radioiodinated Hyp is mainly taken up by the liver and quickly eliminated via bile with a short biological half-life, minimizing hepatobiliary injuries and preventing the risk of renal damage, which is often seen with radioiodinated antibodies or peptides.

To extend the application scope, NTRT could be suitable to improve other necrosis-induced therapies, such as thermal ablation, transcatheter embolization, external beam radiotherapy and most systematic molecular targeting treatments. For instance, radiofrequency ablation (RFA) has become a standard treatment for liver cancer, renal cancer, thyroid cancer and some malignant bone tumors. One problem of RFA is the heat sinking effect that protects the tumor cells near vessels from thermal damage, leading to tumor recurrence [37]. Regarding the central necrosis induced by RFA, NTRT might act as a workable supplement to kill the residual cancer cells and prevent tumor recurrence, thus tackling the heat sinking effect caused by peritumoral blood flow. To further develop this novel approach of NTRT, other species of animals such as canines, swine or nonhuman primates will be used to verify the modality's safety and efficacy. For application of the NTRT in humans, after approval by the ethical committee and regulatory authorities, clinical trials in cancer patients with primary and metastatic solid malignancies, particularly those that failed with existing therapies, will be considered as indications.

There are several limitations in this study. First, the simultaneous implantation of VX2 tumors in the leg and liver may in fact not perfectly mimic the presence of a liver metastasis. Second, whether the approach of NTRT might prolong animal survival was not observed in this study. Third, the stability of the formulation of ^131^I-Hyp in body and toxicity need to be further studied.

In conclusion, ^131^I-Hyp was proven to be a promising necrosis-targeted agent in rabbit VX2 tumor models, with a smaller molecular weight, better *in vivo* stability, higher concentration in necrosis and fast clearance from other organs. As a complementary therapeutic modality, NTRT well improved the anticancer efficacy of VDT with a simple procedure, a high tumor volume control rate and low toxicity, and the procedure could be imaged and monitored by clinical MRI and SPECT suites. The final application of ^131^I-Hyp may provide a new option in our anticancer armamentarium.

## MATERIALS AND METHODS

### Animal model

The protocol for this animal experimentation was approved by the Animal Care and Use Committee of China Medical University. Healthy New Zealand white rabbits weighing 3 kg were provided and maintained by the laboratory animal center of the institute. Fresh VX2 tumors harvested from the tumor-bearing rabbits were cut into 1-mm^3^ blocks, which were inoculated into the right leg and liver of the recipient rabbits using a CT-guided percutaneous puncture implantation method [38]. The rabbits were maintained for 2 weeks to develop solitary lesions with a diameter of approximately 2 centimeters in both the liver and right leg. The tumor models were evaluated by magnetic resonance imaging (MRI).

### Anesthesia

The rabbits were anesthetized by intramuscular injection of 3% soluble pentobarbitone (0.5 mg/kg; Sigma, St. Louis, MO, USA) for tumor implantation procedures and *in vivo* imaging examination.

### Drug preparation and administration

Combretastatin A4 phosphate (CA4P), 5-[(2R)-2-hydroxy-2-(3,4,5-trimethoxyphenyl)ethyl]-2-methoxyphenol (Sigma, St. Louis, MO, USA), as a typical VDA, was diluted in phosphate-buffered saline (PBS) at a concentration of 10 mg/ml and intravenously injected at 10 mg/kg of body weight [38, 39].

Hypericin, 4,5,7,4′,5′,7′-hexahydroxy-2,2′-dimethylnaphthodianthrone (with a purity 99%; Biopurify Phytochemicals Ltd., Chengdu, China; http//www.biopurify.com) was diluted in dimethyl sulfoxide (*DMSO,* Sigma, St. Louis, MO, USA) at 1 mg/ml and radioiodinated by ^131^I (Na^131^I, HTA Co., Ltd., Beijing, China) using an iodogen-coating method (a standard electrophilic substitution reaction) [15, 22]. Briefly, by using an Iodogen (1,3,4,6-tetrachloro-3α,6α-diphenylglycouril) tube (Molde, Haimen Jiangsu, China), radioiodination was conducted by adding 900 MBq of Na^131^I, 50 μl of 0.5 M phosphate buffer solution (pH 7.4), and then 2mg of Hyp. The mixture was incubated for 20 minutes, and the reaction was terminated by removal of the reaction mixture. The labeling rate was determined by ascending paper chromatography using Xinhua Filter paper grade No. 1 and 0.01 N HCl. Then the agent was intravenously injected at a dose of 74 MBq/kg.

### Study protocol

Thyroid was blocked in all rabbits by feeding Lugol's solution (5% iodine and 10% potassium iodide mixed in distilled water; total iodine content of 126.5 mg/mL) from 3 days before the experiments. Twenty-seven tumor-bearing rabbits were divided into 3 groups randomly (9 rabbits in each group). The animals in each group received sequential intravenous injections at a 24-hour interval. Animals in Group A (blank control group) received the two solvents (PBS and DMSO). Animals in group B (VDA control group) received CA4P and DMSO. Animals in group C (NTRT group) received CA4P and ^131^I-Hyp. A 24-h interval between injections allowed the complete formation of necrosis and optimal accessibility to the latter due to partial recovery of tumor vascularization [22-24, 39, 40]. An animal in each group was executed 24 hours after the first injection to verify tumor necrosis induced by CA4P. MRI and SPECT were performed *in vivo* at different time points. All 24 animals with bifocal VX2 tumors (one in the muscle and the other in the liver) in the three groups were executed immediately after the last examination at day 12. Postmortem techniques of histopathology, gamma counting, autoradiography and fluorescence microscopy were used. The study protocol is illustrated in Fig. 2.

### MR imaging

MRI was performed using a 3.0-T MR unit (Signa HDx; General Electric Medical Systems, USA) with a maximum gradient strength of 40 mT/m equipped with a HD Quadrature Knee/Foot Coil. Initially, a T1-weighted (T1W) spin-echo (SE) sequence with axial, coronal, and sagittal images was used as a localizer. Next, enhanced T1W axial images (TR/TE, 200/5.8 ms; FOV, 160×160 mm; imaging acquisition matrix, 256×192) were obtained with a section thickness of 3.0 mm and an intersection gap of 1.0 mm after intravenous bolus injection of gadodiamide (GE Healthcare AS, USA) at 0.1 mmol/kg.

The tumor volume was calculated using a dedicated workstation (Advantage Workstation, ADW 4.5; GE Medical Systems, USA). Using an operator-defined region of interest (ROI), the area of tumor was manually delineated on each tumor-containing slice. The total area of tumor for the slices was calculated after summation and then was multiplied by the slice thickness plus gap to obtain the total tumor volume according to the formula [I] [38, 41]:

Tumor Volume=∑Tumor area on each tumor containing slice×(Slice thickness+Gap) [I]

The tumor doubling time (TDT) was calculated based on the formula [II] [42]:

*TDT = (T-T_0_)×log2 /(logV-logV_0_) [II],* where (T-T_0_) indicates the time interval between two measurements, and V_0_ and V denote the tumor volume at the two points of measurement.

The tumors in the muscle and liver were averaged by groups for tumor volume analysis.

### SPECT imaging

SPECT imaging was performed 2 days and 8 days after injection of ^131^I-Hyp (on day 3 and day 9). Images were acquired using a dual-head SPECT system (Symbia T2; SIEMENS, Malvern, PA, USA) equipped with a high-energy, low-resolution collimator. The imaging acquisition parameters included an energy peak of 340 Kev±20 %, a magnification of 1.00 and a matrix of 128×128. Planar static images were acquired for 50K counts or 5 min, whichever was completed first. Tomographic images were acquired from 30 projections collected for 40 s each over 180° using a 64 × 64 matrix. Data were reconstructed using the iterative mode (SyngoMI 2009; SIEMENS, Malvern, PA, USA). Flash 3D was set to 6. The iteration was 8, and Gaussian was 6 mm. Radioactive hot spots were searched on planar static and tomographic images.

### Measurement of viable rim

After sacrifice of an animal at day 12, the tumor together with its surrounding tissue was resected and swift into the surrounding of −20°C for 60 minutes. Then, the tumor was split into halves along its axial direction. The profiles of the tumor were taken photos digitally and analyzed with Adobe Photoshop CS5 Extended 12.0 (CA, USA). The areas of necrosis and entire tumor were delineated and calculated. Area of viable rim was the difference of area of entire tumor subtracting area of central necrosis, as showed by formula [III].

$${\mathrm{Area}}_{\mathrm{viable}\;\mathrm{rim}}={\mathrm{Area}}_{\mathrm{entire}\;\mathrm{tumor}}-{\mathrm{Area}}_{\mathrm{central}\;\mathrm{necrosis}}$$

### Gamma counting

Tumor (central necrosis and viable rim), peritumoral tissue (muscle or liver) and other organs (brain, thyroid, heart, lung, kidney, stomach, intestine, colon, bone, blood and skin) were sampled. The samples were weighed separately and then evaluated for radioactivity using an automatic gamma counter (Perkin Elmer; 2470 WIZARD2; USA). Corrections were made for background radiation and physical decay during quantification. The radioactivity of the samples was calculated as the percentage of the injected dose per gram of tissue (ID%/g). Biodistribution of ^131^I-Hyp was expressed by the radioactivity (ID%/g) of different organs at the last time point.

### Autoradiography

The tumor together with its surrounding tissue was resected and prepared as cryostat serial sections. Autoradiography was performed using 10-μm unstained sections with the Molecular Imager FX System (Bio-Rad laboratories, UK). The sections were exposed for 24 hours to a storage phosphor screen, which was then read by a scanner. The acquired autoradiographic images were analyzed using Quantity One 4.3.1 software (Bio-Rad, UK). The intensity of radioactivity was measured by placing a square region of interest (2 mm^2^) on different positions of color-coded images. Next, the sections were stained with hematoxylin and eosin (H&E) for conventional light microscopy.

### Fluorescence microscopy

Fluorescence images were acquired on 5-μm unstained cryostat sections by fluorescence microscopy (Nikon ECLIPSE 80i, Japan) using an excitation wavelength of 510–560 nm (Hyp excited wavelength). The fluorescent intensities of central necrosis, viable rim and peritumoral tissue were measured and analyzed by NIS-Elements D software (Nikon, Japan). Next, the sections were stained with H&E.

### Statistical analysis

Numerical data were expressed as the mean ± standard deviation. Statistical analysis was performed using Graphpad Prism (La Jolla, CA, USA) 5.0 software demo. One-way ANOVA was used to test differences between tumor volumes and TDT among the groups. Unpaired Student's *t* test (two-tailed) was performed to compare the tumor area, intensity of radioactivity and fluorescence intensity between different tissues, and a *p* value less than 0.05 was considered statistically significant.

## Abbreviations

VDT: Vascular disrupting treatment
VDAs: Vascular disrupting agents
Hyp: Hypericin
^131^I-Hyp: Iodine-131-labeled hypericin
NTRT: Necrosis Targeted Radiotherapy
MRI: Magnetic Resonance Imaging
CA4P: Combretastatin A4 phosphate
PBS: phosphatebuffered saline
DSMO: dimethyl sulfoxide
ROI: region of interest
ID%/g: percentage injected dose per gram of tissue
H&E: hematoxylin and eosin
HIF-1á: hypoxiainducible factor 1á
TNT: tumor necrosis treatment
RFA: radiofrequency ablation
